# Overview of Animal Rabies in Kinshasa Province in the Democratic Republic of Congo

**DOI:** 10.1371/journal.pone.0150403

**Published:** 2016-04-07

**Authors:** Augustin Tshibwabwa Twabela, Aaron Simanyengwe Mweene, Justin Mulumbu Masumu, John Bwalya Muma, Boniface Pongombo Lombe, Careen Hankanga

**Affiliations:** 1 Veterinary Laboratory of Kinshasa, Kinshasa, Democratic Republic of Congo; 2 School of Veterinary Medicine, University of Zambia, Lusaka, Zambia; 3 Université Pédagogique National, Kinshasa, Democratic Republic of Congo; Thomas Jefferson University, UNITED STATES

## Abstract

**Introduction:**

Rabies is one of the major public health problems mostly affecting developing countries in Africa and Asia where 99.9% of all rabies related human deaths are recorded each year. In Democratic Republic of Congo, repeated outbreaks have been reported. Despite this, there is little reliable epidemiological data about rabies in the country for the development of effective control strategies.

**Materials and Methods:**

A retrospective study was carried out in Kinshasa Province during a period of five years (2009–2013) to describe the proportion of rabid animals and the species involved in rabies transmission and maintenance. The survey also aimed at describing the spatial-temporal distribution of rabies. To gather information, the daily registers of institutions involved in rabies diagnosis were reviewed and each rabies case was traced back to area of occurrence for collection of geographic coordinates.

**Results and Discussion:**

A total of 5,053 attacks were registered involving six animal species including dog, cat, monkey, rabbit, rat, and pig. Based on clinical observations, rabies was reported in dogs and cats while data obtained from the laboratory confirmed rabies cases included dogs, cats and a goat. The annual distribution showed a significant decrease of rabies cases from 2009 up to 2011 and a later increase up to 2013. There was no difference in rabies occurrence between seasons (p = 0.721). Rabies cases were three times higher in peri-urban zone than in urban zone OR = 3.4 (95% CI: 2.3–5.1). The positive proportion of rabies was 2.6% (95% CI: 2.1–3) based on clinical evidence and 65.9% (95% CI: 50–79.5) for laboratory confirmed cases.

**Conclusion and Suggestion:**

This study confirms the endemicity of rabies in Kinshasa where occurrence of rabies cases was related to human population density and lifestyle. In order to control rabies, there is need to set up a surveillance program and implement efficient mass vaccination campaigns of susceptible animals.

## Introduction

Rabies is a severe, progressive and incurable viral encephalitis [1] caused by highly neurotropic viruses of the *Lyssavirus* genus and the *Rhabdoviridae* family [2,3]. Although a wide range of mammals are susceptible and can transmit rabies [4], the Order of C*arnivora* such as domestic dogs (*Canis lupus*), raccoons (*Procyon lotor*), skunks (*Spilogale putorius*), foxes (*Vulpes vulpes*), jackals (*Canis aureus*) and the Order of *Chiroptera* (bats) are considered as reservoirs [5], where the domestic dogs represent urban reservoir; jackals, foxes and raccoons are the principle sylvatic reservoirs [6]. All over the world, 55,000 human deaths are reported each year due to rabies [7] from the bites inflicted by rabid domestic dogs [8]. Developing countries, especially Africa and Asia, account for 99.9% of this mortality [9]. Much of the rabies problems experienced in Africa and Asia result from lack of effective control strategies and epidemiological surveillance systems [10]. Controlling rabies requires sound knowledge of epidemiological parameters such as the species involved in the disease maintenance and transmission, the spatial-temporal distribution and incidence or prevalence [10].

It has been argued that the biggest challenge to evaluate the impact of rabies as well as planning and assessing the control measures in a particular area is lack of information about its incidence [10]. It has also been observed that in most of the developing world, passive surveillance mechanisms are ineffective; this makes rabies to be under-reported and consequently, its impact is certainly not well appreciated [11].

In Kinshasa, the capital of the Democratic Republic of Congo (DRC), repeated rabies outbreaks have been reported in the last decade with human deaths [12,13]. Despite this situation, there is no enough documented epidemiological information on the disease upon which to base the development of effective control strategies.

Therefore, this study aimed at assessing the epidemiological situation of rabies in Kinshasa for a period of five years (2009–2013) in order to provide relevant information required for the formulation of appropriate control strategies.

## Materials and Methods

### Study area

Kinshasa Province, the capital of DRC is located at -4.3250 latitude and 15.3222 longitude. According to the Köppen classification, the climate in Kinshasa is Aw_4_ type [14]. Kinshasa is bordered to the East by Bandundu Province, to the South-West by Bas-Congo Province and to the North by the Congo River. Some of these boundaries constitute mainly bushy areas and forests that provide sanctuaries to different wild animals.

**Fig 1:**
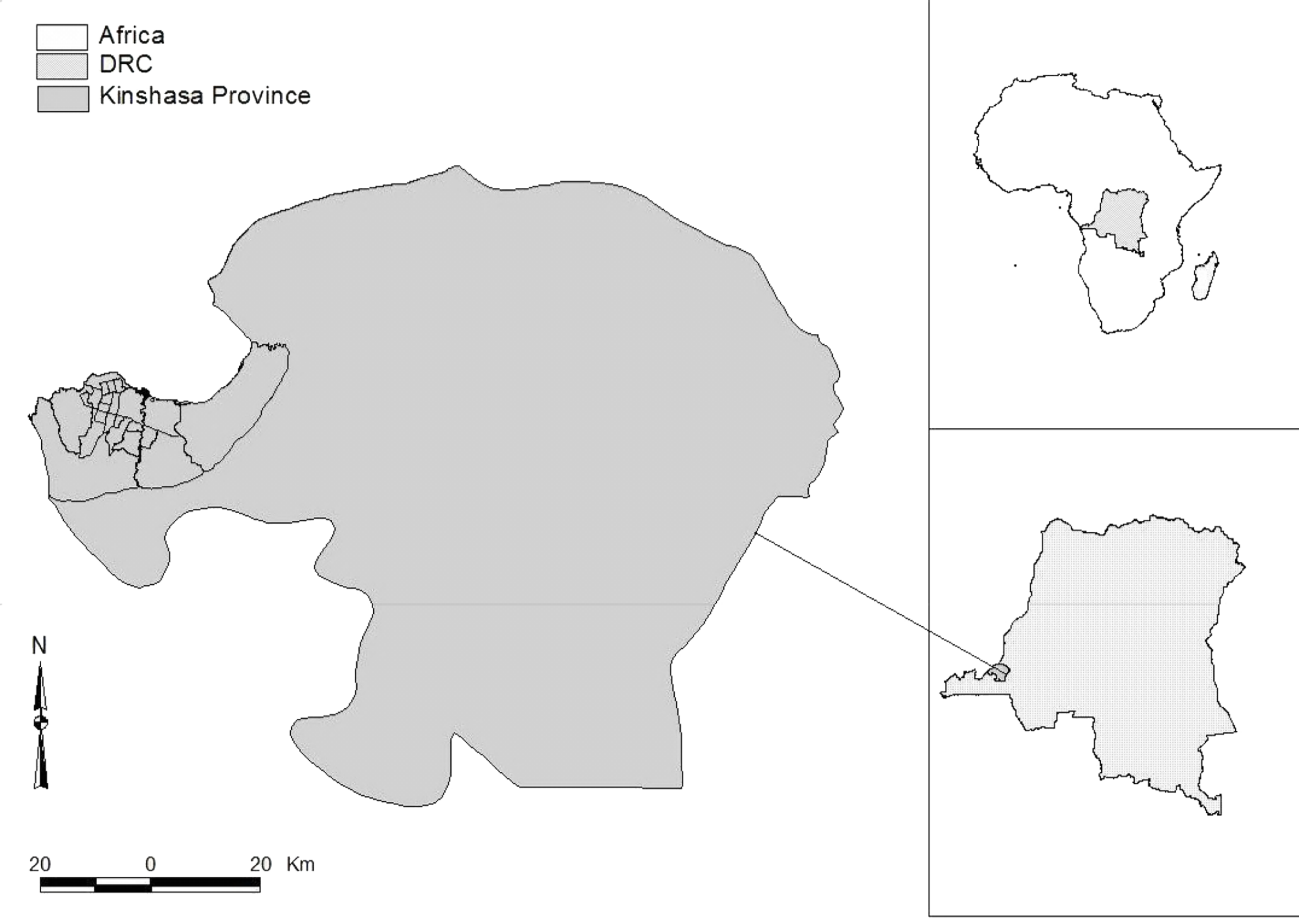
The location of Kinshasa Province and its 24 communes.

Kinshasa covers an area of 9,965 km^2^ and is divided into 24 communes grouped in three zones including urban, peri-urban and rural zone (Fig 1). In 2008, the human population of Kinshasa was estimated at 8,000,000; at least 65% were concentrated in six communes including Ngaliema, Mont-Ngafula, Kimbaseke, Masina, Nsele, Kisenso, Ndjili and Selembao [15] which constitute the biggest part of the peri-urban zone. In the same year, according to the Office for Rabies vaccination and control (OVCR), the dog population was estimated at 72,000, which represented almost one dog per 100 persons (1/100).

Depending on the lifestyle, people keep and consider dogs in different ways: in the urban areas, dogs are often kept on a leash or in kennels. However, in the peri-urban areas, dogs can be kept on a leash or in kennels (family dogs); owned but never been kept on leash or in kennels (neighbourhood dogs);, and unowned and move freely (stray dogs). In the rural zone, dogs are never kept on a leash or kennel, and the dog population size tends to be small because of the low human population density. These rural dogs often serve as hunting dogs. Cats have been increasing in number in the recent years. They are kept by households to control mice and rats. Some cats also live as synanthropic animals and often move freely between households.

### Data source and collection

Being a legal disease in DRC, only public institutions are allowed to deal with rabies. Thus, four institutions including veterinary clinic of Ngaliema, OVCR at the veterinary clinic of Gombe, the Institut National de Recherche Biomédicale (INRB) and the Veterinary Laboratory of Kinshasa (LaboVetKin) served to gather information.

The lack of human post exposure prophylaxis (PEP) centres in the health care structures in Kinshasa has promoted these two veterinary clinics to serve as human PEP centres. This makes these public institutions to be reference centres in Kinshasa for rabies matters.

The daily registers of these institutions were reviewed to collect information on rabies. As most of the cases observed in the clinics were not submitted to Laboratories for confirmation, this made laboratory cases to be independent from clinical cases.

Thus in this study, a rabid animal was either: (a) a confirmed case as an animal that was diagnosed positive at the laboratory using the Direct Fluorescent Antibody Test (DFAT) for rabies [2] or (b) a clinical case as an animal that showed pathognomonic signs of rabies such as behavioural change, agitation/aggressiveness, hydrophobia, paralysis and died [16] or killed during the observation period without laboratory confirmation. All identified rabies cases (a) and (b) were then traced back to the source of occurrence and geographic coordinates were taken at the addresses provided in the registers. The collected information was entered in an Excel® data base and coded according to type of variables.

### Data analysis

The descriptive statistic was performed using SPSS software version 20 to describe the distribution of rabies cases. Fisher’s Exact and Chi square tests were used to appreciate the occurrence of rabies cases within variables. Rabies cases distribution was mapped using the ArcView GIS^®^ 3.2 software from the geographic coordinates collected for each rabies case.

### Ethical approval

Ethical clearance to collect rabies data from Government Institutions handling both human and animal data was obtained from the Inspector of the Provincial Division of Agriculture, Fisheries and Livestock, who is the custodian of rabies data in the province of Kinshasa. Since our study did not involve contact with human patients, a commitment of confidentiality was made with the Inspector of the Provincial Division of Agriculture, Fisheries and Livestock to protect the rights of the rabies victims whose records were obtained.

## Results and Discussion

### The proportion of rabies cases

A total of 5,053 attacks were recorded in the veterinary clinics that inflicted either bite or scratch to humans and other animals. Of these, 128 cases 2.5% (95% CI: 2.1–3%) were noted to be rabid based on clinical observation. Nevertheless, from 44 brain samples received and examined at the laboratories (LaboVetKin and INRB), 29 were found positive to rabies, making 65.9% (95% CI: 46–82%) as positive proportion of the laboratory submitted specimens.

The difference observed between the two proportions was due to the fact that for the clinical cases, in the denominator the big number of all suspected cases reported after an attack reduced the proportion of rabid cases. In the other hand, the confirmed cases were only some animals with pathognomonic signs of rabies that were brought to the laboratory for diagnosis and often independent of cases observed in the veterinary clinics that swelled the proportion of laboratory cases.

Since these proportions were calculated from data recorded under a passive reporting system, underestimation of the actual burden is likely. It assumed that the positive proportion of both the clinical and confirmed cases could be higher if an active surveillance system was applied. Such a system is likely to capture the maximum number of rabies cases in the population.

In addition, it was observed that among the 5,053 attacks registered, 83 (1.6%) animals were killed and 15 (0.3%) disappeared just after attack without a follow-up or a veterinary observation. In these categories, some animals could have been rabid giving raise to the rabies cases. Such unrecorded rabies cases could lead to disease under-reporting thereby giving wrong perception of the true status of rabies in Kinshasa Province.

### Species involved in rabies transmission and maintenance

Out of the 5,053 attacks registered throughout the five years reviewed, six animal species were involved including dog, cat, tamed monkey, rat, rabbit and pig. Based on clinical observation, dogs comprised 119 (93%) cases and cats 9 (7%) cases were found rabid (Table 1).

**Table 1:** The species involved in attacks and the clinical cases of rabies registered in veterinary clinics between 2009 and 2013.

Species	Number of attacks reported	Clinical cases observed
Dog	4,750 (94%)	119 (92.9%)
Cat	225 (4.4%)	9 (7%)
Monkey	40 (0.79%)	0
Rat	33 (0.65%)	0
Pig	3 (0.05%)	0
Rabbit	1 (0. 19%)	0
**Total**	**5,053**	**128 (2.5%)**

For the laboratory confirmed cases, out of 29 brain samples received at the laboratories, three species were positive: dog 27 (93.1%) cases, cat 1(3.4%) and goat 1 (3.4%) case.

Rabies in dogs and cats was established based on both clinical and laboratory diagnosis. The study revealed that in Kinshasa, dogs were the main carrier and transmitter of domestic rabies followed by cats as observed in Ethiopia [17] and Kenya [11]. In the case of Kinshasa, this situation was due to the fact that dogs and cats were the most domesticated animals for different purposes. These animals were often kept free-ranging and their management not well mastered. In such a population, the circumstances were favourable for promotion of circulation and maintenance of rabies virus.

Although the aim was to describe the animal rabies, three clinical cases of human rabies was reported. Cases of rabid humans biting their relatives even though the transmission of human-to-human is rare and not well documented [3]. Fortunately, the bitten persons received the full Post Exposure Prophylaxis (PEP) on time and did not develop rabies.

### The spatial distribution of rabies

During the period under review, the occurrence of rabies was unevenly distributed in the 24 communes of Kinshasa. Clinical rabies was observed in 20 communes; four of the communes had a highest rate (15 to 38 cases), four more communes were moderately affected (4 to 8 cases) and 11 others were less affected (1 to 3 cases).

However, confirmed rabies was reported only in nine communes: Ngaliema (10 cases), Mont-Ngafula (6 cases), Lemba (6 cases), Kimbaseke (2 cases), Selembao, Kitambo, Gombe and Limete (1 case) each.

**Fig 2:**
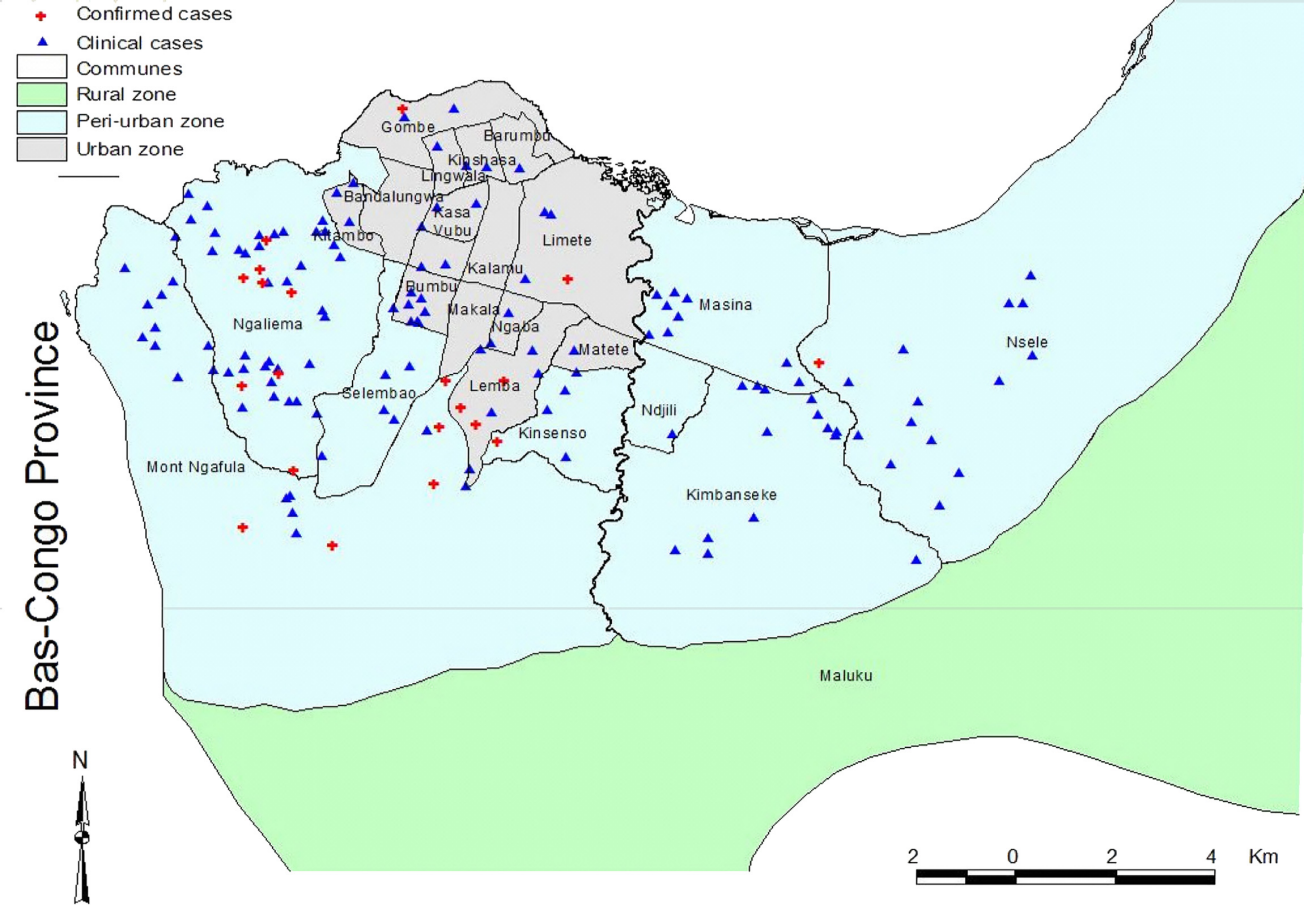
The spatial distribution of clinical and confirmed rabies cases in 3 zones of Kinshasa from 2009 to 2013.

As shown in the Fig 2, both clinical and confirmed rabies were concentrated in the peri-urban zone. Rabies occurrence was three times higher in the peri-urban zone compared to urban zone OR = 3.4 (95% CI: 2.3–5.1) for the clinical cases and twice for the confirmed cases OR = 1.94 (95% CI: 0.53–7.01). However, there was no rabies case reported in the rural zone.

The high rate of rabies in the peri-urban zone could be explained by the high human demography in the area that enhanced the number of pets kept by humans, which facilitated the circulation and transmission of rabies among the pets and humans as well. In comparison to the urban zone, the human population was less for different reasons such as limitation of accommodation, expensive lifestyle. In the rural zone which was almost a farm land with very low human population density resulting in few pets. This resulted in reduced risk of exposure to rabies.

Within the peri-urban zone, Ngaliema commune was the most affected followed by Mont-Ngafula (Fig 2). This could be explained by the fact that these two communes were close to Bas-Congo Province where many rabies cases have been reported in the past [12]. It could also be due to their close proximity to bushy areas where Jackals (*Canis aureus*) were suspected to carry and transmit wild rabies to domestic dogs as observed in Tanzania [18].

### The temporal distribution of rabies

#### The annual distribution

**Fig 3:**
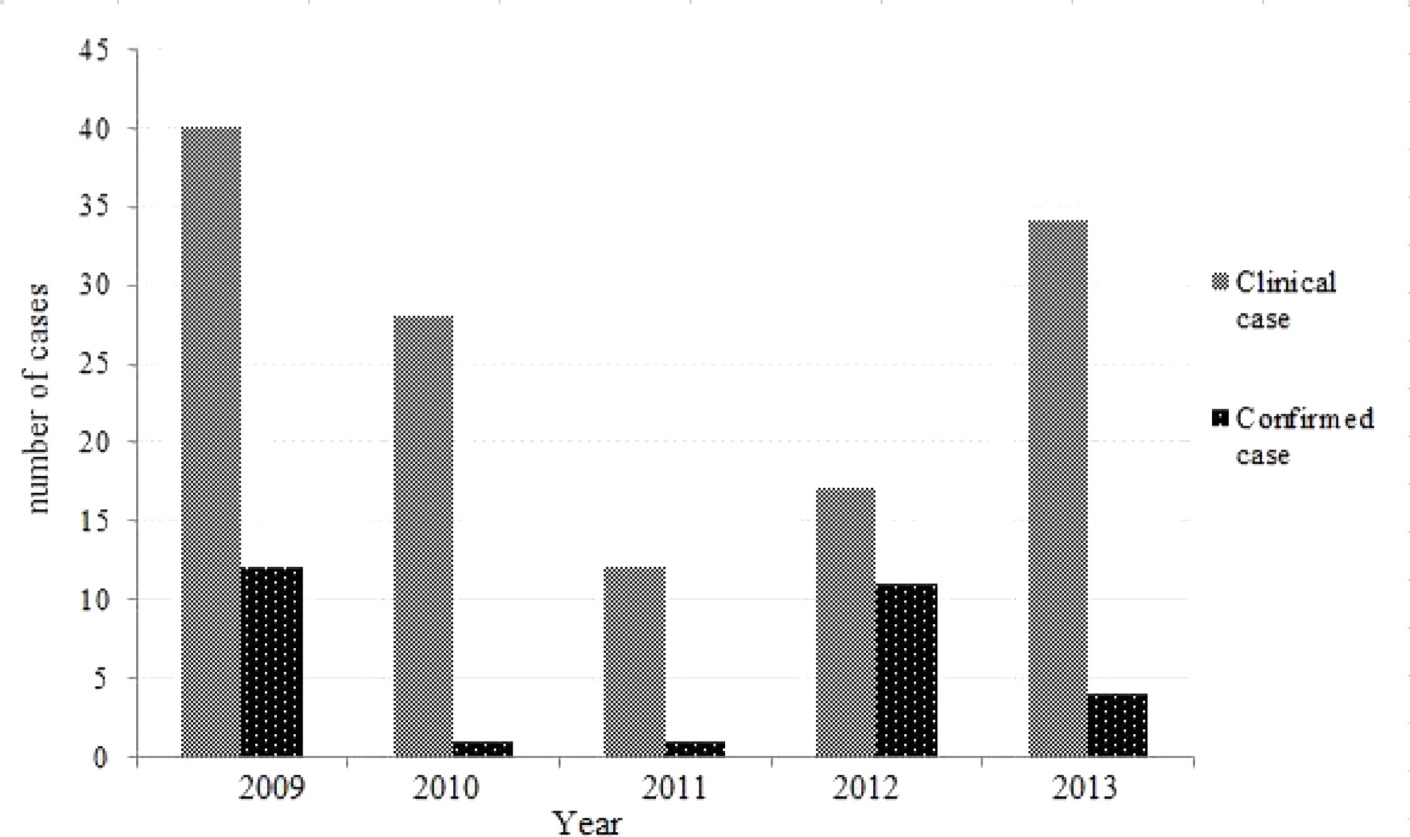
Annual distribution of clinical and confirmed rabies cases in Kinshasa from 2009 to 2013.

The study showed that there was a difference in the occurrence of clinical rabies cases during the five years (p = 0.034). The highest number of cases was reported in 2009 and decreased in 2010 up to 2011 and then increased in 2012 up to 2013 (Fig 3). The laboratory cases gave almost the same trend as the clinical cases even if the number of case is less.

This graphic situation (Fig 3) has an inverted curve, especially for clinical cases, where the observed depression in the number of cases could be attributed to the mass vaccination campaign in the two of the most affected communes (Ngaliema and Mont-Ngafula) after the occurrence of rabies outbreaks a few years earlier and in 2009 The immunisation of animals reduced the number of susceptible pets and, accordingly, stopped the rapid spread of the rabies virus [19]. As the mass vaccination was done just once in 2009, after one year the animals’ immunity waned. In addition, the naïve population of newly born dogs and cats raised the number of susceptible animals as stated by Gsell and colleagues [20]. This could explain the constant diminution of rabies cases in 2010 and 2011 and a sudden increase in 2012 up to 2013.

For the confirmed rabies, there was a raise in the number of cases in 2009 probably due to the same situation of outbreaks which raised awareness among the people to bring suspected samples to the laboratory for analysis. But with the quiescent period following mass vaccination campaigns, no samples were brought to the laboratory, until the number of rabies cases began to increase following a decrease in dog population immunity.

#### The monthly and seasonal distribution

There was no difference observed for rabies occurrence in months either for clinical cases (p = 0.113) or confirmed cases (p = 0.1) throughout the study period. After grouping months in term of seasons, no difference was observed for clinical cases (p = 0.721) and confirmed cases (p = 0.213).

Our observation was different from a similar study conducted in Zambia in which more rabies cases were recorded during the cold period (June to August) [21]. In that study, authors attributed this as to coincidence with the mating season during which dog-fights tend to increase because of the cool temperature which allowed dogs to increase their activities and being able to move for long distances from their habitual places. While in hot season, dogs tend to avoid many activities and preferred to rest in cold places. Nevertheless, comparing to Kinshasa, the difference of temperature between the “cold period” in the dry season and other months in the year was not very distinct. This could induce dogs’ activities remaining constant throughout the year and the season may not have influenced rabies occurrence.

## Conclusion

The findings of this study proved that rabies is endemic in Kinshasa where dogs and cats played a major role as carriers and transmitters of the rabies virus. The months or seasons did not influence the occurrence of rabies. The peri-urban zone with high human population concentration was the most affected with rabies.

It was further observed that the mass vaccination campaign applied once in selected areas reduced rabies occurrence. Therefore, this strategy, if well planned, and in combination with disease surveillance could be useful in controlling rabies in Kinshasa Province as well as in the entire country. The difference observed in the number of clinical cases registered and the samples taken to the laboratories for analysis revealed further a lack of collaboration between the field practitioners and laboratories that led to underreporting of rabies in Kinshasa. We suggest that this collaboration should be strengthened and include all disciplines considered as key players involved in rabies control. Since passive surveillance could lead to an underestimation of rabies cases, active surveillance should be implemented to evaluate the real epidemiological situation of rabies in Kinshasa.
